# A Message from the Editor-in-Chief

**Published:** 2020-12-23

**Authors:** R. Jankauskas

**Affiliations:** “A Message from the Editor-in-Chief”, Acta medica Lituanica, 27(2), p. 45. Available at: https://www.journals.vu.lt/AML/article/view/21932 (Accessed: 15April2021).

## Abstract

A Message from the Editor-in-Chief

This issue of Acta medica Lituanica is the result of further evolution: from now on, our journal is being published by the Vilnius University Press. Probably you noted changed design, but most changes are within the system of submission and publishing. All they serve the main goal – wider and more effective dissemination of knowledge.

Vilnius University Press is the academic community owned publishing platform – it means that academics play a key role in dissemination of results of their research. This enables also more effective implementation of academic freedom, more innovations, more transparency. Finally, it also means more Open Science. Publications in our journal are and will be freely accessible and free of article processing charges.

This is already the second issue that was formed at the time of extreme challenges for biomedical community due to the global COVID-19 pandemic. Many of us found ourselves at the frontline dealing with this critical situation and fulfilling our duty to society. This issue starts with a review paper dedicated to the problem No.1; some more are under review. Another paper is dealing with professional burnout – not a minor problem for health professionals nowadays. Other research papers and case studies remind us about life before and (let’s hope) after the pandemic.

Finally, I want to thank our reviewers who sacrificed their time evaluating manuscripts:

Sigita Aidietienė

Vidmantas Alekna

Karolis Ažukaitis

Valdas Banys

Aušra Beržanskytė

Žana Bumbulienė

Jolanta Dadonienė

Gytė Damulevičienė

Edvardas Danila

Audrius Dulskas

Milda Endzinienė

Eglė Ereminienė

Žymantas Jagelavičius

Ligita Jančorienė

Ričardas Janilionis

Augustina Jankauskienė

Dalius Jatužis

Tomas Kačergius

Vytautas Kasiulevičius

Jacek Kubica

Vaidutis Kučinskas

Zita Kučinskienė

Limas Kupčinskas

Sigita Lesinskienė

Mieczyslaw Litwin

Rūta Mameniškienė

Rūta Nadišauskienė

Alvydas Navickas

Janina Petkevičienė

Narūnas Porvaneckas

Tomas Poškus

Dainius Pūras

Rūta Sargautytė

Jūratė Šipylaitė

Povilas Sladkevičius

Eugenijus Stratilatovas

Arūnas Strumila

Rasa Strupaitė-Šileikienė

Virgilijus Tarutis

Vytautas Tutkus

Albertas Ulys

Vincas Urbonas

Algirdas Utkus

Birutė Vaišnytė

Respectfully,

Prof. Dr Rimantas Jankauskas,

Editor-in-Chief

Acta medica Lituanica

